# Cryptic Bumblebee Species: Consequences for Conservation and the Trade in Greenhouse Pollinators

**DOI:** 10.1371/journal.pone.0032992

**Published:** 2012-03-09

**Authors:** Paul H. Williams, Jiandong An, Mark J. F. Brown, James C. Carolan, Dave Goulson, Jiaxing Huang, Masao Ito

**Affiliations:** 1 Department of Entomology, The Natural History Museum, London, United Kingdom; 2 Institute of Apiculture, Chinese Academy of Agricultural Sciences, Beijing, China; 3 School of Biological Sciences, Royal Holloway University of London, Egham, Surrey, United Kingdom; 4 Department of Zoology, School of Natural Sciences, Trinity College Dublin, Dublin, Ireland; 5 Department of Biology, National University of Ireland Maynooth, Maynooth, County Kildare, Ireland; 6 Biological & Environmental Sciences, University of Stirling, Stirling, United Kingdom; 7 Higashi-Ku, Sapporo, Japan; Institut Mediterrani d'Estudis Avançats (CSIC/UIB), Spain

## Abstract

Commercial greenhouse growers in both Japan and China are increasingly using reared orange-tailed bumblebees known previously as *Bombus hypocrita* Pérez as pollinators. Phylogenetic analysis of the DNA (COI) barcodes with Bayesian methods shows that this “species” is a long-standing confusion of two cryptic species. We find that the orange-tailed bumblebees in North China are actually part of the widespread Russian (otherwise white-tailed) *B. patagiatus* Nylander (as *B. patagiatus ganjsuensis* Skorikov, **n. comb.**), whereas the orange-tailed bees in Japan are true *B. hypocrita*. This situation has been further complicated because two other cryptic species from North China that were previously confused with the Russian *B. patagiatus* are now recognised as separate: *B. lantschouensis* Vogt **n. stat.** and *B. minshanensis* Bischoff **n. stat.**. As demand for pollination services by greenhouse growers inevitably increases, these bees are more likely to be transported between countries. In order to conserve genetic resources of pollinator species for their option value for future food security, we advocate preventing trade and movement of *B. patagiatus* from China into Japan and of *B. hypocrita* from Japan into China.

## Introduction

Commercial movement of species outside of their natural ranges has had significant negative impacts on biodiversity [1]–[3]. Consequently, such translocation is now regulated at national and international levels [4]. To be effective, regulation depends upon accurate taxonomy to identify the relevant species. Here we show that imprecise taxonomy poses a substantial threat to ecologically important pollinators.

Bumblebees are among the most important pollinators in wild ecosystems, but recently have been suffering worldwide declines [5], [6]. At the same time, bumblebees have become increasingly important commercially for their pollination services to agriculture, especially for tree fruits, berries, and greenhouse crops such as tomatoes [7]. This has led to the widespread movement of bumblebees between countries to provide pollination services, an industry now worth billions of dollars annually [8]–[10]. Such commercial translocation has resulted in the introduction and invasion of exotic bumblebee species (and their pathogens) into New Zealand [11], Tasmania [12], South America [13], [14], and Japan [15], [16].

The situation in Japan has been particularly well-studied. There, *Bombus terrestris* (Linnaeus) was introduced from Europe into greenhouses, but feral colonies were soon discovered and the species has shown not only invasive spread within Japan, but is also replacing the indigenous *B. hypocrita* Pérez in many areas [15], [16]. One solution is the commercial development of indigenous pollinators and in Japan, *B. hypocrita* is undergoing trials [17]. In China, government-funded research projects have been established to study the feasibility of what has been understood to be the same species [18]–[20]. *B. hypocrita* is currently recognised as an orange-tailed species, believed to be distributed in both Japan and China [18], [21]–[27]. Alongside these bees in China, some other bumblebees with white tails are also being used as greenhouse pollinators [28] and many of these have been widely referred to previously using the name *B. patagiatus* Nylander [18], [22], [25], [26], [29]. But just as *B. hypocrita* in Japan is currently seriously threatened by introduced *B. terrestris* (Linnaeus), so populations of all of these bumblebees in Japan and China could be threatened by introductions between countries if the bumblebees used are in fact not conspecific.

All of these commercially important species belong to the subgenus *Bombus s. str.* and many of these species are well known for being cryptic in Europe [30]. Not all individuals can be identified with confidence using morphological characters [31] and specialists also disagree among themselves on the precise criteria for diagnosing them [32]. Nonetheless, support for the interpretation that there are separate species continues to grow from studies of morphology [33], enzyme electrophoresis [34], [35], male labial gland secretions [36], [37], and DNA sequences [38]–[41]. However, this European work has been done against a background of very patchy knowledge of the Asian species of the group.

In this paper we use DNA barcodes to show that the orange-tailed bumblebees previously recognised as *B. hypocrita* in Asia are actually comprised of parts of two more geographically restricted species: *B. hypocrita*, and a part of *B. patagiatus* with an unrecognised cryptic colour pattern. Given that these bees are already being used for pollination in greenhouses in Asia, we discuss the consequences of our results for conserving the genetic resources of these commercially valuable pollinators and the need to restrict movement of bumblebees between China and Japan.

## Materials and Methods

### Sampling bees

We sampled bumblebees as part of a review of all of the species of the subgenus *Bombus s. str.* across their entire global distributions [42], which encompass most of the northern hemisphere. Progress with the taxonomy of this group using only morphological evidence has been difficult [31], but recently new insights have been gained by using DNA-sequence data [38], [40]. In insects, sequences of the mitochondrial COI (*cox-1*) gene have been used to discover species identical to those recognised by more traditional methods [43]. Inheritance of mitochondrial genes is reasonably well understood [44]. It avoids the problems with nuclear genes that arise from having multiple alleles, while the absence of indels makes alignment for homology straightforward. Mitochondrial genes also have a relatively high substitution rate, so that even the short COI ‘barcode’ region of 658 nucleotides can be used to distinguish the most closely-related taxa [45], [46], and COI-barcode sequences as short as 100 nucleotides can be diagnostic for 90% of the species in other animal groups [47]. Consequently, despite many potential pitfalls and the desirability of external supporting evidence, COI barcodes can be useful for recognising likely cryptic species [48]. Permissions for collection and export of bumblebee specimens from China were obtained via the CAAS Institute of Apiculture and CAS Institute of Zoology, number 2010-86 issued 15.ix.2010.

### DNA-barcode data

Most specimens were extracted, amplified, and sequenced at the Biodiversity Institute of Ontario, University of Guelph, as part of the BEE-BOL campaign to barcode the bees of the world [49]. COI-barcode extraction, amplification, and sequencing used the standard protocols described by Hebert *et al.* [45]. Universal primers for the COI-barcode sequence for insects were used (variants *LepF1* and *LepR1* [50]). COI-barcode sequences (without primer sequences) from the samples were aligned using the ClustalW function within BioEdit (version 7.0.9.0; www.mbio.ncsu.edu/BioEdit/bioedit.html, accessed 2010) and trimmed to a common frame length of 658 nucleotides that is shared by most samples. Overall, we analysed 559 sequences from 279 localities in 33 countries from throughout the world-wide range of the subgenus. For the analysis reported here, sequences for species that are the focus of this paper (i.e. *B. hypocrita*, *B. patagiatus*, and cryptic relatives: 106 sequences) were reduced to 20 unique haplotypes using Collapse (version 1.2; darwin.uvigo.es/software/collapse.html, accessed 2011) after ranking sequences by their length. For the species that are not the focus of this analysis, we used sequences from among the samples available that were collected closest to the species' type localities (details of sequence data are given in Appendix S1).

### Phylogenetic analysis

Phylogenetic relationships among sequences were estimated using MrBayes (version 3.1.2 [51], [52]), from 10 million generations of the metropolis-coupled Markov-chain Monte Carlo (MCMC) algorithm with four chains, chain temperature set to 0.2, and with sampling of the trees every 1000 generations. We found the nucleotide-substitution model that fitted our COI-barcode data best according to jModelTest (version 0.1.1 [53]) to be the general time-reversible model with a gamma frequency distribution of changes among sites and allowing invariant sites (GTR+G+I). Burn-in was set initially to 10%, with convergence between two separate runs of the analysis judged to have occurred when the average standard deviation of the split frequencies approached stationarity. Post burn-in stability of the log likelihood of the cold chain was confirmed using Tracer (version 1.5.0 [54]) and stability of the sample groups was confirmed using AWTY (version 0.8.1 [55]). The sample of 16,002 post burn-in trees from both replicates was combined. Trees were rooted using data for the sister groups of *Bombus s. str.* as sequences from *B. (Pyrobombus) vagans* Smith, *B. (Alpinobombus) alpinus* (Linnaeus), and *B. (Al.) balteatus* Dahlbom following the results of Cameron *et al.* [56] from a phylogenetic analysis of five genes across almost all bumblebee species. MEGA (version 4.0 [57]) was used to generate intra- and inter-group genetic distances and to determine the number of informative polymorphisms that support the phylogenetic grouping of the cryptic taxa and the recognition of new species. Genetic distances (sequence divergences) were calculated using the Kimura two-parameter (K2P) distance model [58]. The translated amino acid sequences were also compared to determine whether polymorphisms were non-synonymous. Treatment of taxon names follows the rules laid down by ICZN [59].

## Results

**Figure 1:**
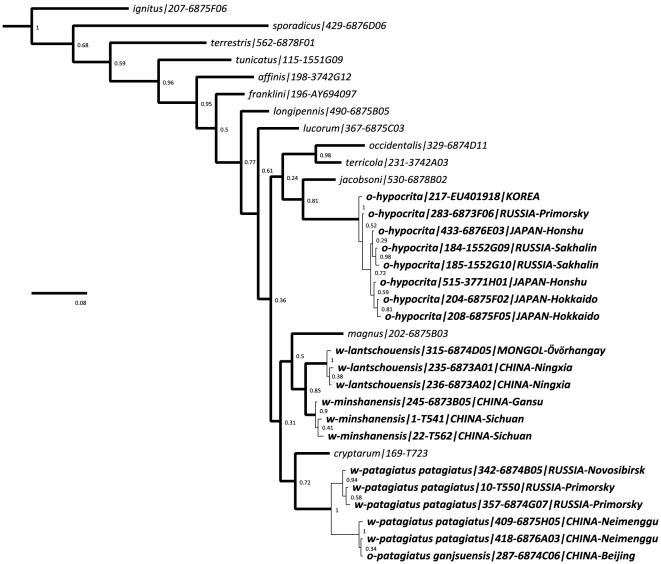
Estimated Phylogenetic Tree for Asian Bumblebees Resembling *B. hypocrita* and *B. patagiatus*. Consensus Bayesian tree for (o) orange- and (w) white-tailed examples representing the unique haplotypes from among 106 COI-barcode sample sequences of *B. hypocrita, B. lantschouensis, B. minshanensis* and *B. patagiatus*, together with examples representing all of the other species of *Bombus s. str.* (from a total of 559 sequences). The codes following the taxon names are the specimen identifiers from the project database and from BOLD (or from other external databases including GenBank), followed by country and province. Values next to the nodes are posterior probabilities for groups (groups with values of <0.9 are considered unreliable) and the scale bar represents 0.08 substitutions per nucleotide site.

Our estimate of phylogenetic relationships from COI sequences (Figure 1) shows only weak support (posterior probabilities) for several of the species groups, including especially those representing the relationships of *B. hypocrita* and *B. magnus* Vogt with other species. However, this tree shows strong support for the monophyly of the groups interpreted here as the species *B. hypocrita, B. lantschouensis* Vogt, *B. minshanensis* Bischoff, and *B. patagiatus*. It does not support a monophyletic group of orange-tailed bees (o) among the white tailed bees (w) within our focal group of *B. hypocrita* and *B. patagiatus* in the former broader sense. In contrast, among these bees it supports: (1) that there are two relatively distantly related groups of Asian orange-tailed bees - one group in Japan, Korea, Primorsky, and Sakhalin (*B. hypocrita*) and another group in North China (*B. patagiatus ganjsuensis* Skorikov); and (2) that the white-tailed bees considered previously to be a single species, *B. patagiatus*, are comprised of at least three distinct groups (*B. lantschouensis*, *B. minshanensis*, and *B. patagiatus*).

The recognition of *B. hypocrita*, *B. lantschouensis*, *B. minshanensis*, and *B. patagiatus* as separate species is supported by a number of unique and diagnostic polymorphisms (Table 1). Fifteen, four, and five unique polymorphisms are present in COI barcodes for each of *B. hypocrita* (8 haplotypes), *B. lantschouensis* (3 haplotypes), and *B. minshanensis* (3 haplotypes), including non-synonymous polymorphisms. Within-group K2P distances for these species are low, ranging from 0.002 to 0.004 (Table 2). *B. patagiatus* is characterised by seven unique polymorphisms, although a clear divergence exists within this group, as indicated by the high intra-group distance value of 0.014 (synonymous polymorphisms). Two subgroups are recognised: Group 1, of haplotypes from white-tailed bumblebees from Russia and the far north east of China; and Group 2, of haplotypes from both white-tailed and orange-tailed bumblebees from North China. Analyses of these groups separately showed intra-group distances of 0.002 (Group 1) and <0.001 (Group2). The latter value equates to just one polymorphism among the 42 specimens and three haplotypes of *B. patagiatus* found in this study. Eleven unique polymorphisms are shared by the COI barcodes for *B. patagiatus* Group 2 (Table 1), providing strong support for the cryptic status of the orange-tailed *B. patagiatus ganjsuensis* and the placement of these orange-tailed bumblebees as part of *B. patagiatus* and not *B. hypocrita*.

**Table 1:** COI-Barcode Polymorphisms among Haplotypes of *B. patagiatus* and *B. hypocrita*.

		Nucleotide position												
Tail colour	Taxon	19	43	49	82	85	112	133	196	247	293	295	302	308
white	*B. patagiatus patagiatus* haplotypes 1–3	C	T	A	T	T	T	G	C/T	T	C	T	A	A
white	*B. patagiatus patagiatus* haplotypes 4–5	C/T	C	A	C	T	C	A	T	C	T	A	C	T
orange	*B. patagiatus ganjsuensis* haplotype 1	C	C	A	C	T	C	A	T	C	T	A	C	T
orange	*B. hypocrita* haplotypes 1–8	C	T	A/C	T	C	T	A	C	T	T	A	C	A

		Nucleotide position												
		346	373	420	449	479	505	520	541	547	562	598	625	631
white	*B. patagiatus patagiatus* haplotypes 1–3	**A**	A	T	**A**	**C**	**T**	C/T	C	T	**A**	**C**	**G**	**G**
white	*B. patagiatus patagiatus* haplotypes 4–5	**A**	A	T	**A**	**C**	**T**	C	T	A	**A**	**C**	**G**	**G**
orange	*B. patagiatus ganjsuensis* haplotype 1	**A**	A	T	**A**	**C**	**T**	C	T	A	**A**	**C**	**G**	**G**
orange	*B. hypocrita* haplotypes 1–8	T	C/T	A/C	G	T	C	T	T	T	T	T	A/G	A

Bold nucleotides show diagnostic characters with respect to other species of *Bombus s. str.* (Figure 1).

**Table 2:** COI-Barcode Characteristics for *B. hypocrita*, *B. lantschouensis*, *B. minshanensis*, and *B. patagiatus*.

Taxon	No. of individuals	No. of haplotypes	No. of unique polymorphisms	Intra-group distances	Inter-group distances					
*B. hypocrita*	16	8	11	0.005		*hyp*	*lan*	*min*	*pat* Grp 1	*pat* Grp 2
*B. lantschouensis*	37	3	4	0.002	*hyp*	-				
*B. minshanensis*	11	3	4	0.002	*lan*	0.06	-			
*B. patagiatus* Group 1	23	3	3	0.002	*min*	0.04	0.05	-		
*B. patagiatus* Group 2	19	3	7	<0.001	*pat* Grp 1	0.04	0.05	0.02	-	
*B. patagiatus* (Groups 1+2)	42	6	7	0.014	*pat* Grp 2	0.02	0.06	0.03	0.03	-

Genetic distances are calculated using the Kimura two-parameter (K2P) distance model [58]. Numbers of unique polymorphisms from comparisons of all four species and the sister-taxa *B. magnus* and *B. cryptarum* (Figure 1).

## Discussion

Taxonomic revisions of bumblebees and other commercially important insect groups are needed in part to prevent people from inadvertently transporting cryptic non-native species. Such revisions crucially need to be global in scope, both to include all of the known species as well as to include broadly representative samples from across the entire breadths of all of the species' geographic ranges. Attempts to revise groups from more restricted geographic sampling have led to problems. For example, the name *B. minshanicola* Bischoff has recently been used [60] for a ‘neglected taxon’ of *Bombus s. str.* from China. This name does not appear in our Figure 1 because we agree from a global analysis [42] with earlier studies [61] (see also [33]) that recognised *B. minshanicola* from Gansu as part of the broader species *B. longipennis* Friese, which extends further west within China and the Himalaya. While we also note that there are many potential pitfalls when using COI barcodes for exploring relationships among taxa [62], there is as yet no evidence that these have affected studies of bumblebees [60], [63].

### Disambiguation of species

**Figure 2:**
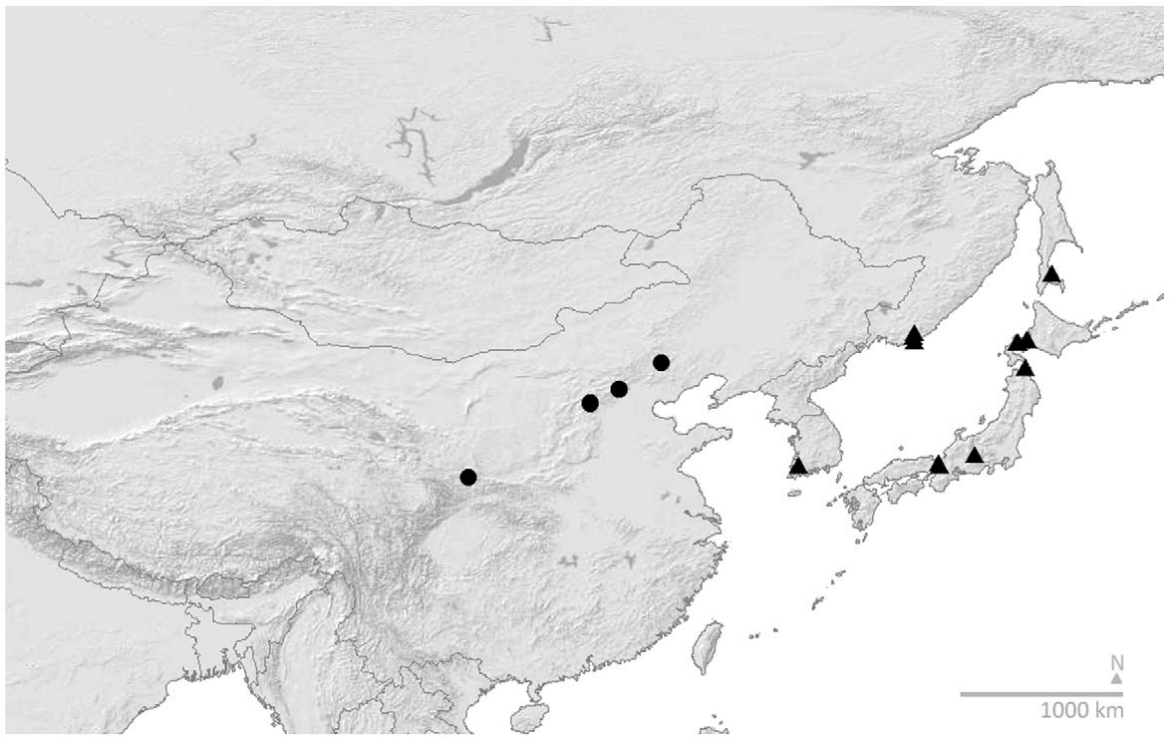
Orange-Tailed *B. hypocrita* Sensu Tkalcu Recognised as *B. patagiatus ganjsuensis* and *B. hypocrita*. Distribution of the orange-tailed taxon *B. hypocrita* in the sense of Tkalcu [21], revised from the results in Figure 1 to two species: black spots, *B. patagiatus ganjsuensis*; and black triangles, *B. hypocrita*. Records shown include only specimens identified in our data from COI barcodes. Map showing shaded relief and international boundaries, Cartesian orthonormal projection.

Figure 2 uses our sequenced samples to map the former concept of *B. hypocrita*, as revised by Tkalcu [21] (map on his page 89) as a taxon of orange-tailed bumblebees, distributed from Gansu to Japan. From our COI-based estimate of relationships (Figure 1), we recognise two species within Tkalcu's [21] broader concept in Fig. 2: *B. hypocrita* in Japan and adjacent Pacific provinces of Asia (syntypes examined, Muséum National d'Histoire Naturelle, Paris), and a second species in North China. For the latter Chinese bumblebees, the oldest available name is *Bombus ikonnikovi ganjsuensis* Skorikov [64] (holotype examined, Zoological Institute of the Russian Academy of Sciences, St Petersburg), revised here to *Bombus patagiatus ganjsuensis* Skorikov (new combination).

**Figure 3:**
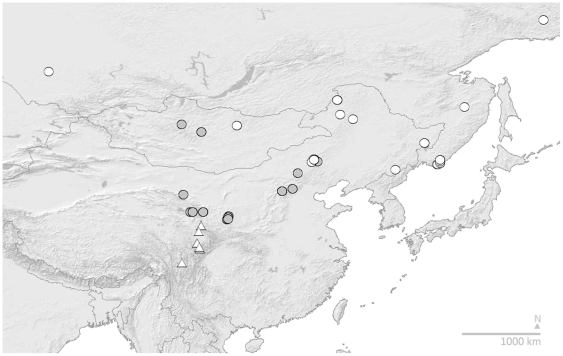
White-Tailed *B. patagiatus* Sensu Tkalcu Recognised as *B. patagiatus patagiatus*, *B. lantschouensis*, and *B. minshanensis*. Asian distribution of the white-tailed taxon *B. patagiatus* in the sense of Tkalcu [29], revised from the results in Figure 1 to three species: white spots, *B. patagiatus patagiatus*; grey spots, *B. lantschouensis*; and white triangles, *B. minshanensis*. Records shown include only specimens identified in our data from COI barcodes. Map showing shaded relief and international boundaries, Cartesian orthonormal projection.

Similarly, Figure 3 maps the former concept of *B. patagiatus*, as revised by Tkalcu [29] (figure on his page 52) as a taxon of white-tailed bumblebees, distributed from near the Finnish border of Russia in the west to Sachalin in the east, and then south westwards through North China to Gansu and Sichuan. From our COI-based estimate of relationships (Figure 1), we recognise three species within Tkalcu's [29] concept in Fig. 3: *B. patagiatus patagiatus s. str.* (type presumed lost [29], although the taxon concept is not in doubt), *B. lantschouensis* (new status, syntypes examined, Zoölogisch Museum der Universiteit van Amsterdam), and *B. minshanensis* (new status, syntypes examined, Museum für Naturkunde an der Humboldt-Universität, Berlin). *B. lantschouensis* and *B. minshanensis* are reciprocally monophyletic with non-synonymous polymorphisms and no individuals with intermediate colour patterns are known to us, so we recognise them as separate species for the first time. Assessment of evidence from DNA barcodes has uncovered multiple cryptic species in other insects [45], [48], suggesting that this situation could be widespread among insects.

**Figure 4:**
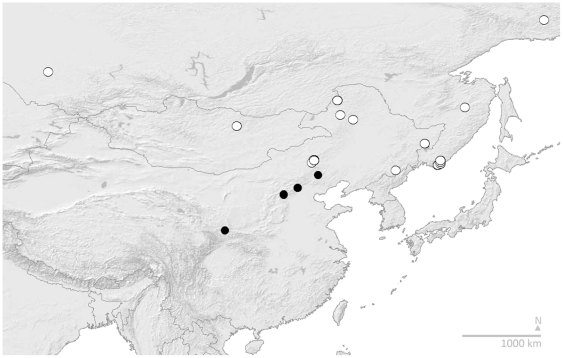
Asian Distribution of the Revised Concept of *B. patagiatus*. These bees are revised from the results in Figure 1 to two subspecies: white spots, white-tailed *B. patagiatus patagiatus*; and black spots, orange-tailed *B. patagiatus ganjsuensis*. Records shown include only specimens identified in our data from COI barcodes. Map showing shaded relief and international boundaries, Cartesian orthonormal projection.

In consequence, our revised concept of *B. patagiatus* (Figure 4) reunites white-tailed bees (open circles) from the Taiga forests of Russia to the north of the Mongolian desert, with orange-tailed bees (black spots) previously misidentified as *B. hypocrita*, from the hills of North China on the southern side of the Mongolian desert. During specially-targeted fieldwork in 2010, specimens were also collected from an isolated patch of suitable habitat on a mountain top (near Huanggangliang) in between these two regions, within the barrier zone formed by the Mongolian arid belt (in Neimenggu or Inner Mongolia). These individuals (our Group 2) all have the COI haplotype of *B. patagiatus ganjsuensis*, but have either varying degrees of intermediate colour patterns with narrow dorsal pale bands (like *B. patagiatus ganjsuensis*) and white tails (like *B. patagiatus patagiatus*), or even have broad dorsal pale bands and white tails resembling *B. patagiatus patagiatus* (our Group 1). Therefore, while the orange-tailed colour pattern appears to be restricted to the south of the Mongolian arid zone, closely related haplotypes (Group 2) and individuals with intermediate colour patterns extend into suitable habitats within the arid zone. We recognise them all here as parts of one species for the first time.

### Movement of cryptic commercial pollinators between countries

Our results are significant because conserving the genetic resources of commercially important pollinator species for their option value for future food security in a changing world means that we need to consider the potential consequences of moving bumblebees and their pathogens outside their indigenous ranges and into new contact with other species. It is already known that introductions of *B. terrestris* into Japan have resulted in the spread of feral populations, the growth of which has coincided on a local scale with declines in populations of indigenous *B. hypocrita* [15], [16]. Therefore with due regard to the precautionary principle [5], [65], we advocate preventing any movement of *B. patagiatus* from China into Japan (where there are no indigenous populations of *B. patagiatus*) and of *B. hypocrita* from Japan into China (where *B. hypocrita* is, as far as we know, absent) until it can be proven safe. We also recommend research to assess the potential of the indigenous species for use in commercial pollination in greenhouses within each region. Deliberate movement of bumblebee species between countries will otherwise become more likely as increasing demand for food puts pressure on commercial growers to deliver reliable pollination in ever larger areas of greenhouse crops. Critically, minimising the risk from commercial movement of species and their pathogens (to which different bumblebee populations may have differing resistance, even within a species) depends upon a full understanding of their taxonomy.

## Supporting Information

Appendix S1 Accession numbers for sequence data for the samples used in Fig. 1, including IDs from the BOLD database.
(DOC)
