# Supplementary material for: Cryptic Bumblebee Species: Consequences for Conservation and the Trade in Greenhouse Pollinators
Source: PLoS One. 2012 Mar 9;7(3):e32992. doi: 10.1371/journal.pone.0032992 (PMC3302899; doi:10.1371/journal.pone.0032992)
Supplement: Appendix S1 — Accession numbers for sequence data for the samples used in Fig. 1, including IDs from the BOLD database. (DOC) [file pone.0032992.s001.doc]

**Appendix S1** Accession numbers for sequence data for the *Bombus* samples used in Fig. 1, including IDs from the BOLD database (boldsystems.org), project BOBO.

| Taxon | BOLD number | GenBank number | Country | Province | Sex/caste |
| --- | --- | --- | --- | --- | --- |
|  |  |  |  |  |  |
| *B. ignitus* | 6875F06 |  | Japan | Kyushu | worker |
| *B. sporadicus* | 6876D06 |  | Sweden | Dalarna | worker |
| *B. terrestris* | 6878F01 |  | Sweden | Uppsala | queen |
| *B. tunicatus* | 1551G09 |  | Nepal | Karnali | queen |
| *B. affinis* | 3742G12 |  | Canada | Ontario | worker |
| *B. franklini* | — | AY694097 | USA | Oregon | male |
| *B. longipennis* | 6875B05 |  | China | Xizang | worker |
| *B. lucorum* | 6875C03 |  | Sweden | Uppsala | male |
| *B. occidentalis* | 6874D11 |  | USA | Washington | worker |
| *B. terricola* | 3742A03 |  | Canada | Northwest Ter. | male |
| *B. jacobsoni* | 6878B02 |  | India | Kashmir | male |
| *B. hypocrita* | — | EU401918 | South Korea | — | — |
| *B. hypocrita* | 6873F06 |  | Russia | Primorsky | queen |
| *B. hypocrita* | 6876E03 |  | Japan | Honshu | worker |
| *B. hypocrita* | 1552G09 |  | Russia | Sakhalin | queen |
| *B. hypocrita* | 1552G10 |  | Russia | Sakhalin | queen |
| *B. hypocrita* | 3771H01 |  | Japan | Honshu | queen |
| *B. hypocrita* | 6875F02 |  | Japan | Hokkaido | queen |
| *B. hypocrita* | 6875F05 |  | Japan | Hokkaido | male |
| *B. magnus* | 6875B03 |  | UK | Scotland | queen |
| *B. lantschouensis* | 6874D05 |  | Mongolia | Övörhangay | worker |
| *B. lantschouensis* | 6873A01 |  | China | Ningxia | worker |
| *B. lantschouensis* | 6873A02 |  | China | Ningxia | worker |
| *B. minshanensis* | 6873B05 |  | China | Gansu | worker |
| *B. minshanensis* | T541 |  | China | Sichuan | worker |
| *B. minshanensis* | T562 |  | China | Sichuan | male |
| *B. cryptarum* | T723 |  | Denmark | Jutland | queen |
| *B. patagiatus patagiatus* | 6874B05 |  | Russia | Novosibirsk | worker |
| *B. patagiatus patagiatus* | T550 |  | Russia | Primorsky | worker |
| *B. patagiatus patagiatus* | 6874G07 |  | Russia | Primorsky | worker |
| *B. patagiatus patagiatus* | 6875H05 |  | China | Neimenggu | worker |
| *B. patagiatus patagiatus* | 6876A03 |  | China | Neimenggu | worker |
| *B. patagiatus ganjsuensis* | 6874C06 |  | China | Beijing | male |
|  |  |  |  |  |  |
